# External snapping hip syndrome is associated with an increased femoral offset

**DOI:** 10.1007/s00590-021-03123-9

**Published:** 2021-09-22

**Authors:** Octavian Andronic, Stefan Rahm, Benjamin Fritz, Sarvpreet Singh, Reto Sutter, Patrick O. Zingg

**Affiliations:** 1 Department of Orthopaedics, Balgrist University Hospital, University of Zurich, Forchstrasse 340, 8008 Zurich, Switzerland; 2 Department of Radiology, Balgrist University Hospital, University of Zurich, Forchstrasse 340, 8008 Zurich, Switzerland

**Keywords:** External snapping hip, Greater trochanteric pain syndrome, Coxa saltans, Trochanteric bursitis

## Abstract

**Background:**

External snapping hip syndrome (ESH) is postulated to be one of the causes of greater trochanteric pain syndrome, which also includes greater trochanteric bursitis and tendinopathy or tears of the hip abductor mechanism. However, it was not yet described what kind of bony morphology can cause the snapping and whether symptomatic and asymptomatic individuals have different imaging features.

**Purpose:**

It was the purpose of this study to look for predisposing morphological factors for ESH and to differentiate between painful and asymptomatic snapping.

**Methods:**

A consecutive cohort with ESH and available magnetic resonance imaging (MRI) between 2014 and 2019 was identified. The control group consisted of patients that underwent corrective osteotomies around the knee for mechanical axis correction and never complained of hip symptoms nor had undergone previous hip procedures. The following parameters were blindly assessed for determination of risk factors for ESH: CCD (corpus collum diaphysis) angle; femoral and global offset; femoral antetorsion; functional femoral antetorsion; translation of the greater trochanter (GT); posterior tilt of the GT; pelvic width/anterior pelvic length; intertrochanteric width. Hip and pelvic offset indexes were calculated as ratios of femoral/global offset and intertrochanteric/pelvic width, respectively. For the comparison of symptomatic and asymptomatic snapping, the following soft-tissue signs were investigated: presence of trochanteric bursitis or gluteal tendinopathy; presence of surface bony irregularities on trochanter major and ITB (Iliotibial band) thickness.

**Results:**

A total of 31 hips with ESH were identified. The control group (n = 29) consisted of patients matched on both age (± 1) and gender. Multiple regression analysis determined an increased hip offset index to be independent predictor of ESH (r =  + 0.283, *p* = 0.025), most likely due to the higher femoral offset in the ESH group (*p* = 0.031). Pearson correlation analysis could not identify any significant secondary factors. No differences were found between painful and asymptomatic snapping on MRI.

**Conclusions:**

A high hip offset index was found as an independent predictor for external snapping hip in our cohort, mainly due to increased femoral offset. No imaging soft-tissue related differences could be outlined between symptomatic and asymptomatic external snapping.

**Level of evidence III:**

This journal requires that authors assign a level of evidence to each article. For a full description of these Evidence-Based Medicine ratings, please refer to the Table of Contents or the online Instructions to Authors  www.springer.com/00590.

## Introduction

Coxa saltans refers to snapping hip and involves three main locations, extra-articular (either external or internal) or intra-articular. The most common form of coxa saltans is the external extra-articular variety which involves either the posterior iliotibial band or the anterior aspect of the gluteus maximus as they move over the greater trochanter during hip flexion and extension or internal and external rotation [1]. It is thought that thickened portions of the posterior iliotibial band or anterior gluteus maximus fibers snap over the greater trochanter causing the catching or “giving way” sensation and inflammation of the trochanteric bursa, which can elicit pain [2, 3].

Due to the distinct anatomic location and often visible snapping, coxa saltans externa is a clinical diagnosis [2]. The snapping of the iliotibial band over the greater trochanter may be observed with the patient lying on the side and palpation of the greater trochanteric region as the hip moves through flexion and extension followed by internal and external rotation [4]. Alternatively, having the patient stand and adduct the hip with circumduction often visibly reproduces the snapping over the greater trochanter [1]. Also, an Ober test [1] can be used to evaluate iliotibial band tightness. In case of clinical doubt, the snapping phenomenon can be observed with dynamic ultrasonography [5].

Coxa saltans externa is postulated to be one of the causes of greater trochanteric pain syndrome, which also includes greater trochanteric bursitis and tendinopathy or tears of the hip abductor mechanism [6]. However, a great number of patients are asymptomatic and do not present any painful symptoms [7].

The pathophysiology of the condition remains uncertain, as to our knowledge, there are only few studies that examined this matter. A study by Krishnamurthy [5] evaluated a small case series where a thickened iliotibial band and focal thickening of the anterior edge of the gluteus maximus muscle could be seen on MRI imaging [5]. Axial T1-weighted images best demonstrated the thickening [5]. Another case report revealed a subluxation of gluteus maximus fibers as a cause of external snapping hip [8].

However, it was not yet described what kind of bony morphology can cause the snapping and whether symptomatic and asymptomatic individuals have different imaging features.

It was the purpose of the current study to evaluate radiographic parameters of bony morphology that might predispose to develop external snapping of the hip, as well as to determine associated findings on MRI imaging. Differentiating between asymptomatic or symptomatic (painful) external snapping was the second main scope of the study.

## Patients and methods

### Demographics of study group

**Fig. 1:**
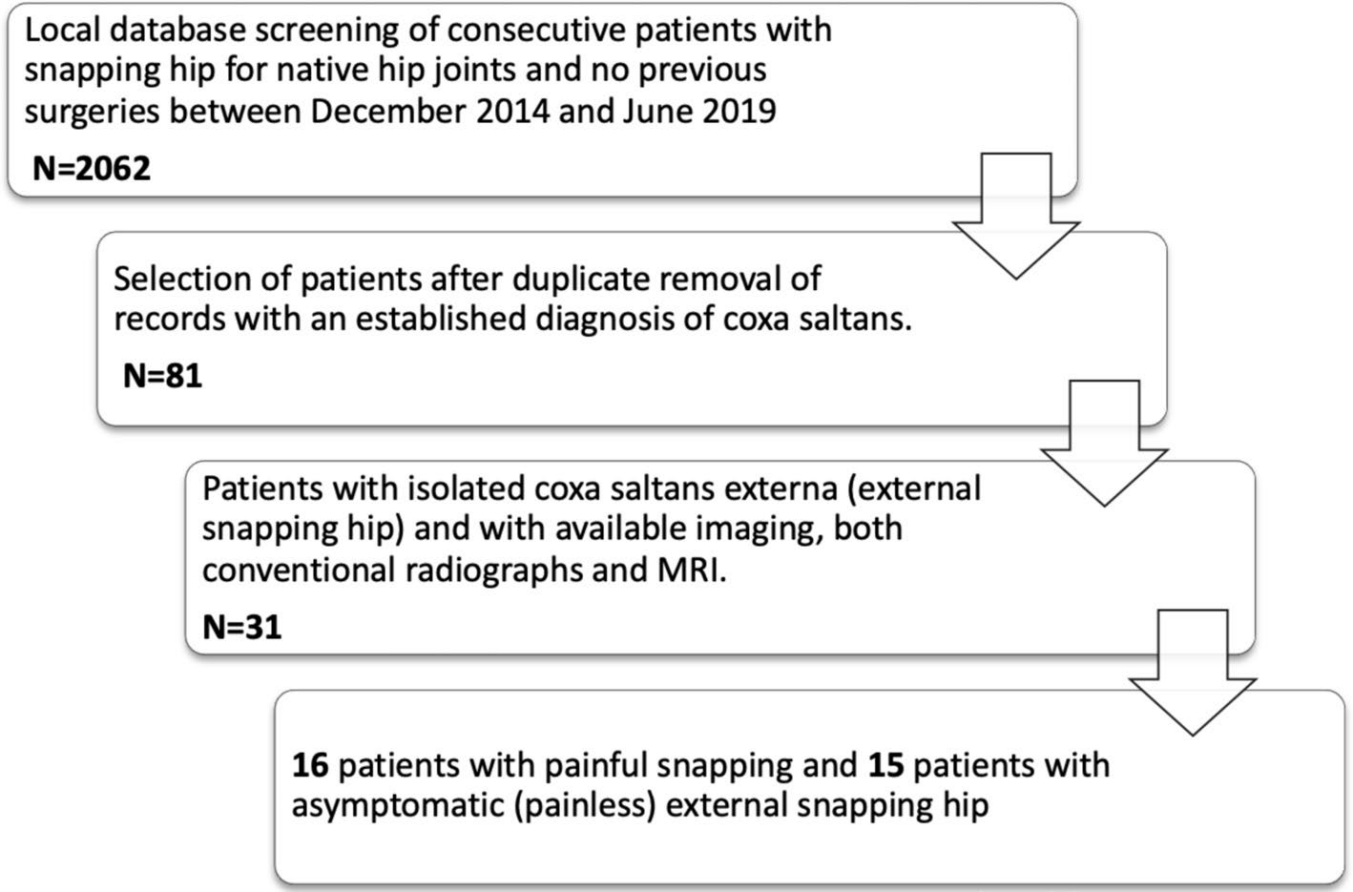
Flowchart of the local database search

Institutional review board approval and of local ethical committee was obtained. All patients that were included signed a written consent form. All clinical records and data from follow-up visits from patients with a clinical diagnosis of external snapping hip were retrospectively extracted from the local database of our institution between December 2014 and June 2019 (Fig. 1). The diagnosis was made by fellowship trained hip preservation consultants through physical examination and by obtaining patients’ medical history. Patients with native hip joints and no previous surgeries without demographic limitations were included only when both conventional radiographs and MRI imaging (3.0 Tesla) with rotational sequences was available. The routine MRI protocol included a 3D water-excitation imaging with steady-state precession gradient-echo sequence that was acquired in a transverse oblique plane parallel to the axis of the femoral neck. Symptomatic external snapping was defined as concomitant pain during the snapping phenomenon.

### Matched control group

For the control group, patients with available relevant imaging and asymptomatic hips were extracted from the local database. As such, patients who underwent osteotomies around the knee for correction of frontal plane leg axis deformity (varus/valgus) and never complained of hip symptoms, nor underwent hip surgeries, were included. Patients with available preoperative anteroposterior whole leg radiographs, as well as CT imaging with rotational sequences were further selected. A matched consecutive population on age and gender was extracted from the consecutive cohort between October 2017 and February 2020 to include a total of 29 hips.

### Radiographic parameters

The following parameters were independently and blindly assessed for both groups for determination of risk factors for ESH: CCD (corpus collum diaphysis) angle; femoral and global offset; surface irregularity on the trochanter major (as an indirect sign of abductor pathology); femoral antetorsion; functional femoral antetorsion; translation of the greater trochanter; posterior tilt of the greater trochanter; pelvic width/anterior pelvic length and intertrochanteric distance. In order to adjust the offset distances (mm) which are dependent of patient-specific anatomy, ratios were calculated and compared: “Hip Offset Index” was the ratio between femoral/global offset, while the “Pelvic Offset Index” was the ratio calculated between the intertrochanteric distance and pelvic width.

For comparison between symptomatic (painful) and asymptomatic snapping, the following soft-tissue related parameters were compared between groups: presence of trochanteric bursitis or gluteal tendinopathy and ITB (Iliotibial band) thickness [9].

### Measurement definitions

Surface irregularity on the trochanter major was defined as when pronounced (> 2 mm) changes were visible on conventional radiographs using previous described criteria [10].

**Fig. 2:**
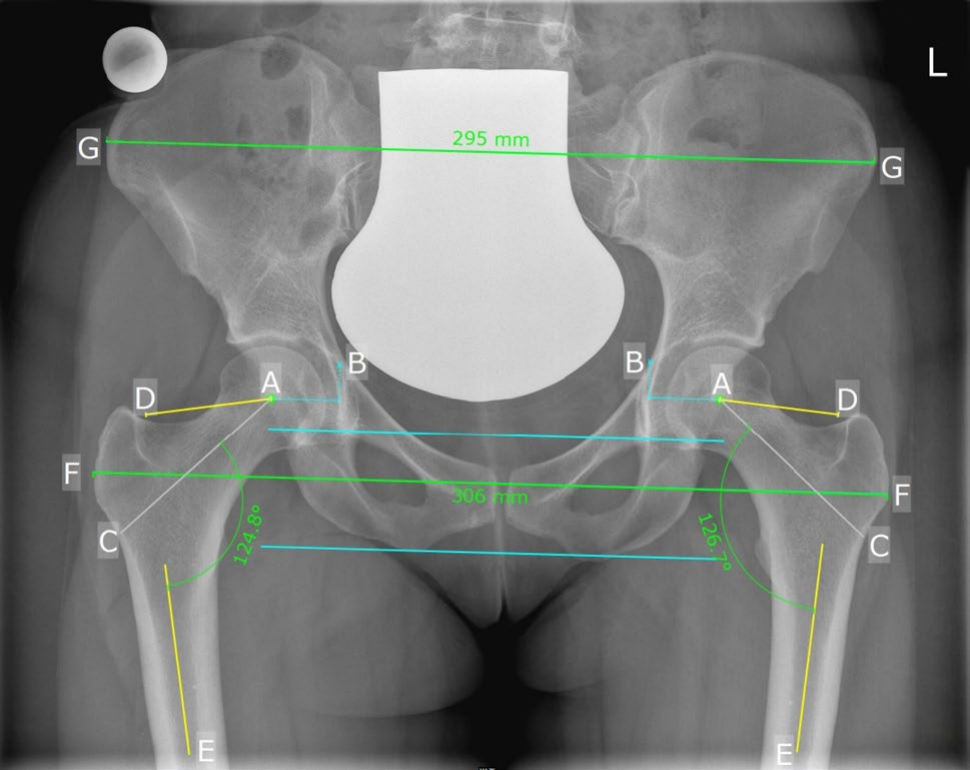
Measurements performed on a posteroanterior pelvic radiograph using reference ball (25 mm): femoral offset (AD) and acetabular offset (AB), CCD angle (between AC and CE), as well as intertrochanteric distance (FF) and pelvic width (GG)

Femoral offset was defined as the distance from the center of rotation of the femoral head to a line bisecting the long axis of the femur [11]. Global offset was calculated as the sum of femoral offset plus the acetabular offset [12, 13], which was the distance between the ilioischial line and the center of rotation of the femoral head (Fig. 2).

Pelvic width or the length of the anterior pelvis, was measured as the greatest distance between the edges of the antero-superior iliac spines (ASIS) [14, 15]. Intertrochanteric distance was also measured on the anteroposterior pelvic radiographs or on full-length orthoradiograms. Full-length orthoradiograms were performed in a sequential basis so no divergence of the X-rays occurs and also no changes in angular values.

Measurements of femoral antetorsion on axial images over the proximal and distal femur were performed according to previously described method on MRI [16, 17].

**Fig. 3:**
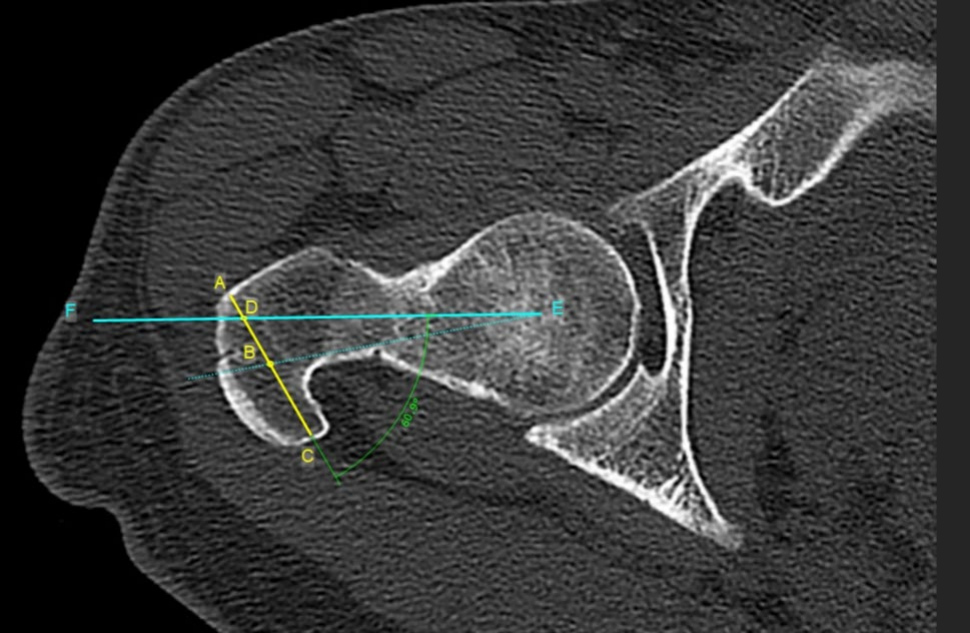
An example measurement of the posterior translation of the GT was defined by the ratio AD/AB according to Batailler et al. Posterior tilt of the greater trochanter (GT), defined as the angle between the femoral neck axis using Murphy’s technique (line EF) and the greater axis of the greater trochanter (line AC). This axis is defined anteriorly by the most lateral point of the anterior facet (point A) and posteriorly by the edge of the GT (point C)** A**—most lateral point of the anterior facet;** B**—center of the axis of the greater trochanter (GT);** C**—edge of the GT;** D**—point of intersection between the femoral neck axis (EF) and GT axis (AC);** E**—center of the femoral head;** F**—marking the direction of the femoral neck axis

**Fig. 4:**
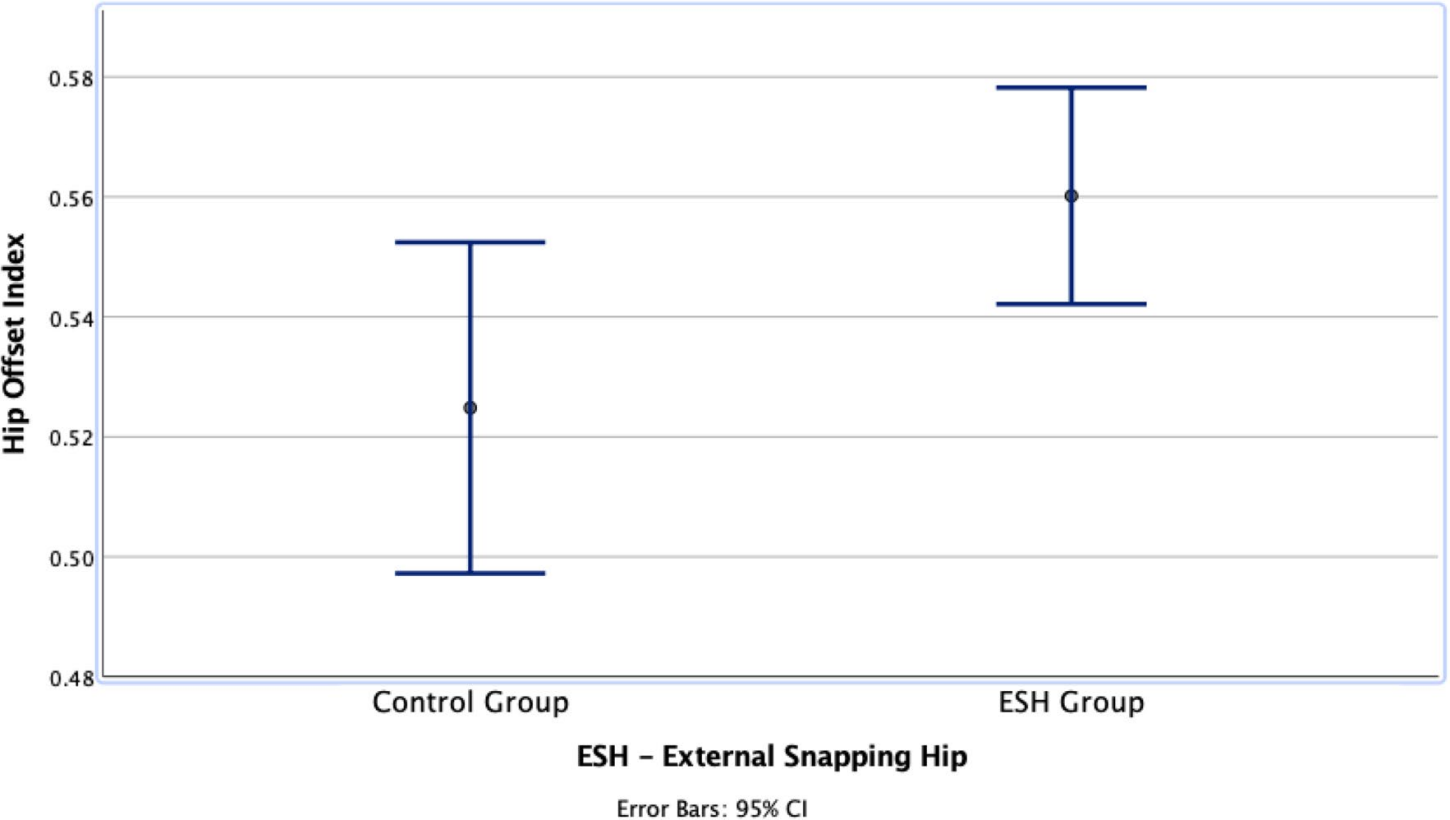
Boxplots representing the comparison of the mean and deviations (95% Confidence Interval) of the hip offset index (femoral/global offset) between the external snapping hip and control groups

Functional femoral antetorsion, posterior translation and tilt of the greater trochanter, were measured used a previously described method by Batailler et al. [18] (Fig. 3). Functional femoral antetorsion was defined as the angle between the posterior femoral condyles and the line joining the center of the femoral head and the middle of the GT’s greater axis (Fig. 3—dotted blue line). Posterior tilt was defined as the angle between the femoral neck axis and the greater axis of the GT. This axis is defined anteriorly by the most lateral point of the anterior facet and posteriorly by the edge of the GT. GT translation was defined by the ratio between the distance from the anterior edge of the axis of the GT to the point of intersection of the femoral neck axis and the axis of the GT—and the distance from the anterior edge of the GT to the center of the axis of the GT (Fig. 3). A lower value of the ratio meaning a more posterior position of the GT compared to the femoral neck axis (Fig. 4).

ITB diameter thickness was measured in the axial plane of MRI examinations. The measurement location was considered as the region of greatest thickness between the most proximal images of the greater trochanter to the most proximal sequence showing the lesser trochanter. [9]

Abductor tendinopathy was defined as presence of tendinosis, calcific tendinitis, partial or full-thickness tears of the gluteus medius and/or minimus tendons [19, 20].

### Statistical analysis

A binary model was constructed due to the dichotomous nature of the dependent variables used (presence of snapping yes/no and presence of symptoms (pain) yes/no) to assess the relationship between association with morphological parameters for external snapping hip between the study and control groups (offset indexes, femoral antetorsion, functional femoral antetorsion, CCD angle, translation and tilt of the greater trochanter) and risk factors for pain in the study group only (abductor tendinopathy, trochanteric bursitis, proximal ITB thickness). A paired Wilcoxon test was used to determine statistical differences between baseline values among compared groups.

An a-priori power analysis for a multiple regression revealed a minimum required sample size of 41 for an anticipated effect size (f^2^) = 0.35 (large effect size) and a desired statistical power of 0.8 (total number of predictors—7). As the compared populations were already matched based on age and gender, no hierarchical analysis was necessary. The multiple regression analysis allowed the inclusion of all covariates in the regression equation for calculation of each risk factor independently to predispose to external snapping: GT translation, GT tilt, femoral antetorsion, functional femoral antetorsion, CCD angle, pelvic offset index as well as hip offset index.

Pearson correlation analysis was performed for comparison of symptomatic versus asymptomatic external snapping hip of the following parameters: presence of tendinopathy, presence of bursitis and IT band thickness. Partial Pearson correlation analysis was used to determine secondary morphological factors after controlling for significant independent risk factors that were found.

## Results

Overall, 60 patients were included in the study. The study group (Fig. 1) comprised of 31 patients. From these, 16 patients presented with painful snapping, while 15 had no pain with the phenomenon. Mean age at first presentation was 25 years (range 16–39, years). There were 8 males and 23 females.

The control group included a total of 29 hips from 29 patients (8 males and 21 females) with a mean age of 25 years (range 15–40, years). As such, the study groups were matched and had no significant differences in terms of age or gender (Table 1).

**Table 1:** Baseline characteristics of compared groups. The p values indicate that values are not statistically different between the two groups (paired Wilcoxon Test)

	Study group (*n* = 31)	Control group (*n* = 29)	Difference
Age, years	25 (range 16–39)	25 (range 15–40)	No (*p* = 0.928)
Gender	8 males, 23 females	8 males, 21 females	
External snapping hip	16 painful external snapping, 15 asymptomatic external snapping	None	
Associated Pathologies	13 FAI; 8 DDH; 6 GTPS	No hip related complaints	

GTPS—clinical diagnosis of greater trochanteric pain syndrome (with or without gluteal tendinopathy); FAI—femoroacetabular impingement; DDH—developmental hip dysplasia

Selected participants from the snapping group had associated pathologies of the hip joint, including femoroacetabular impingement and hip dysplasia (Table 1). However, painful snapping was considered as reproduction of pain only during the snapping phenomenon and not as a chronic state. When comparing the symptomatic (painful) snapping patients with the asymptomatic (painless), Pearson correlation analysis could not identify any soft-tissue parameters that could differentiate between groups: presence of trochanteric bursitis (*p* = 0.86); presence of abductor tendinopathy (*p* = 0.59); iliotibial band thickness (*p* = 0.733) (Table 2).

**Table 2:** MRI soft-tissue findings of patients with external snapping hip syndrome

Parameter	Painful snapping, *n* = 16	Asymptomatic snapping (painless), *n* = 15	Difference (*p* value)
Trochanteric Bursitis	8/16 (50%) of hips	3/15 (20%) of hips	No (*p* = 0.086)
Gluteal tendon (medius/minimus) tendinopathy	8/16 (50%) of hips	6/15 (40%) of hips	No (*p* = 0.59)
Average iliotibial band thickness (range, mm)	4 (3–6 mm)	4 (3–6 mm)	No (*p* = 0.733)

No trochanter surface irregularities were observed in both groups. Multiple regression analysis revealed an increased hip femoral offset/global offset index to be an independent predictor (r =  + 0.283, *p* = 0.025) in patients with external snapping hip (Table 3). This observation (Fig. 4) is most likely based on the significant difference observed in the femoral offset values, which was significantly higher in the ESH group (*p* = 0.03). A Pearson correlation could not reveal any correlation of the femoral torsion (*p* = 0.176) or CCD angle (*p* = 0.874) (Table 4). Greater trochanteric tilt and translation were also not associated with external snapping hip (*p* = 0.383 and *p* = 0.995, respectively).

**Table 3:** Multiple regression analysis and analysis of correlation with each specific morphological predictor

Parameter	Standardized regression coefficient (β)	Statistical significance
CCD angle	0.264	*p* = 0.094
Hip offset index	0.369	***p* = 0.025 (r = + 0.283)**
Pelvic offset index	0.118	*p* = 0.433
Femoral antetorsion	−0.567	*p* = 0.067
Functional femoral antetorsion	0.148	*p* = 0.595
GT posterior tilt	0.198	*p* = 0.254
GT Translation*	0.067	*p* = 0.668

Statistically signifiance bold values are (*p* < 0.05)

*Lower values of GT translation represent a more posterior position of the greater trochanter

“r” value represents the correlation coefficient and may range between −1 and 1. The sign of the coefficient indicates the direction of the relationship, and its absolute value indicates the strength, with larger absolute values indicating stronger relationships

**Table 4:** Pearson correlation analysis with external snapping hip for each predictor

Parameter (Averages ± SD)	Study group	Control group	Significance (coefficient)
CCD angle°	129 ± 5	129 ± 5	*p* = 0.874
Trochanter surface irregularity	0	0	
Femoral offset (mm)	39 ± 5	36 ± 7	***p* = 0.031 (r = + 0.243)**
Global offset (mm)	69 ± 6	68 ± 8	*p* = 0.233
Hip offset index	0.56	0.52	***p* = 0.030 (r = + 0.283)**
Intertrochanteric distance (mm)	301 ± 19	308 ± 19	
Pelvic width (mm)	276 ± 18	287 ± 19	
Pelvic offset index	1.09	1.07	*p* = 0.191
Femoral antetorsion°	15 ± 12	19 ± 10	*p* = 0.176
Functional femoral antetorsion°	25 ± 11	28 ± 9	*p* = 0.418
GT posterior tilt°	68 ± 6	69 ± 7	*p* = 0.383
GT Translation*	0.46	0.46	*p* = 0.995

Statistically signifiance bold values are (*p* < 0.05)

GT—greater trochanter

*Lower values of GT translation represent a more posterior position of the greater trochanter

“r” value represents the correlation coefficient and may range between −1 and 1. The sign of the coefficient indicates the direction of the relationship, and its absolute value indicates the strength, with larger absolute values indicating stronger relationships

## Discussion

As external snapping hip is the most common form of palpable or auditory movement around the hip joints [21], and an often-encountered reason for patients to present in the outpatient clinic, there is an increased interest in sports medicine research to further investigate the aetiology and treatment approach of this condition [22–24]. It was therefore the purpose of this retrospective regression analysis to look for predisposing bony morphological factors that may lead to developing the current condition, as there are no studies to our knowledge that investigated that.

The main finding of our study is that increased femoral/global offset index alone was independently associated with the presence of external snapping hip syndrome, as the regression analysis demonstrated. This has occurred mostly due to the increased femoral offset in the ESH group (*p* = 0.031). We have expected to see a similar finding when comparing the ratio of the intertrochanteric distance/pelvic width (pelvic offset index), that would be more representative of the biomechanical increased distance of excursion of the hip abductors, which may cause snapping. However, this expectation was not confirmed by the results of our study.

Nevertheless, it was already described how femoral offset alone increases the abductor lever arm [11, 25, 26] and therefore probably also the excursion of the soft tissue mass over the GT [27, 28]. The same observation could be determined when talking about femoral neck lengthening, which would also achieve a lateralization of the GT and increase the femoral offset [11, 29], therefore also the abductor lever arm [29]. An increase in the excursion of the iliotibial band can hypothetically occur due to over-tensioning [28] that could lead to iliotibial band friction or snapping. Also, it should be noted that femoral offset can only be underestimated on anteroposterior pelvic radiographs due to variation of femoral torsion or leg rotation [30].

Secondary, we could not find imaging differences between symptomatic and asymptomatic snapping. There was no predictive value in the thickness of the iliotibial band in identifying the symptomatic snapping, as postulated by previous studies [1, 7, 23]. Also, tendinopathy or trochanteric bursitis alone could not predict the presence of pain in our cohort. This could be due to the chronicity of the condition and due to possible differences between occurrence of inflammation (bursitis) and point of time at which the imaging studies were conducted. Another issue that needs to be considered is that the comparison was carried out only within the study group with a reduced overall sample size. Studies using larger patient cohorts are further necessary to elucidate this matter.

Currently, there is a general agreement that external snapping hip should be first treated conservatively [2, 7, 31]. Lewis reviewed the success rates of conservative treatment for snapping hip which ranged between 36 and 67%. Patients reported a reduction in or resolution of symptoms following conservative treatment [7]. Furthermore, an escalation with an ultrasound-guided corticosteroid injection can be considered for a quick pain relief[32] to enable patients to undergo physical therapy [33]. Despite all the above-mentioned efforts, surgical treatment may be necessary when conservative treatment and corticosteroid injection fail [22]. Surgical interventions aim at lengthening of the iliotibial band. Complications associated with surgery have been reported[1, 7] and included abductor weakness, Trendelenburg gait, persistent hip pain, continued snapping, sensory deficit of the lateral femoral cutaneous nerve, painful bursa, and infection [1, 7]. The reasons for failure of conservative treatment are not fully understood [3, 22, 34–37]. Previous studies could not determine prognostic factors that would lead to surgery. Most studies focused on soft-tissue related morphology and did not take into account patient-specific bony anatomy as potential reason for treatment failure [1, 7]. Our contribution to knowledge can therefore not only improve the understanding of the pathophysiology, but may be helpful on investigating the response to conservative treatment.

A limitation of the current study could be the difference between imaging measurements, which were performed slightly differently for the study and control groups: the first on anteroposterior pelvic radiographs and MRI scans, while the second one on orthoradiograms and CT scans. This is, however, known not to be a problem, as the orthoradiograms were sequentially performed with no divergence of X-rays and changes in angular values and because both CT and MRI methods have same levels of reliability and reproducibility for measurement of the proximal femur including femoral version [38].

In conclusion, the current study identifies a high hip offset index (femoral/global offset ratio) as an independent predictor for external snapping hip in our cohort, mainly due to high femoral offset. This may be the base for future studies to investigate its role in the outcome of non-responders to conservative treatment and the need for operative surgery.

## Data availability and material

Data were stored in a local repository RedCap with access provided to the study staff and principal investigator.
